# Effect of municipal biowaste derived biostimulant on nitrogen fate in the plant-soil system during lettuce cultivation

**DOI:** 10.1038/s41598-023-35090-y

**Published:** 2023-05-16

**Authors:** Ferdinando Fragalà, Ivana Puglisi, Elio Padoan, Enzo Montoneri, Piergiorgio Stevanato, Josè Maria Gomez, Natalia Herrero, Emanuele La Bella, Erika Salvagno, Andrea Baglieri

**Affiliations:** 1 Dipartimento di Agricoltura, Alimentazione e Ambiente, Università di Catania, 95123 Catania, Italy; 2 Dipartimento di Scienze Agrarie, Forestali e Alimentari, Università di Torino, 10095 Grugliasco, TO Italy; 3 Department of Agronomy, Food, Natural Resources, Animals and Environment, University of Padua, Padua, Italy; 4 Biomasa Peninsular S.A., Constancia, 38 Bajo, 28002 Madrid, Spain

**Keywords:** Biochemistry, Plant sciences

## Abstract

A main concern of agriculture is to improve plant nutrient efficiency to enhance crop yield and quality, and at the same time to decrease the environmental impact caused by the lixiviation of excess N fertilizer application. The aim of this study was to evaluate the potential use of biopolymers (BPs), obtained by alkaline hydrolysis of the solid anaerobic digestate of municipal biowastes, in order to face up these main concerns of agriculture. The experimental trials involved the application of BPs (at 50 and 150 kg/ha) alone or mixed with different amounts (100%, 60% and 0%) of mineral fertilizer (MF). Three different controls were routinely included in the experimental trials (MF 100%, 60% and 0%). The effect of BPs on lettuce was evaluated by monitoring growth parameters (fresh and dry weights of shoot and root, nitrogen use efficiency), and the N-flux in plant-soil system, taking into account the nitrate leached due to over irrigation events. The activities of enzymes involved in the nitrogen uptake (nitrate reductase, glutamate synthase and glutamine synthase), and the nitrogen form accumulated in the plant tissues (total N, protein and NO_3_^−^) were evaluated. The results show that the application to the soil of 150 kg/ha BPs allows to increase lettuce growth and nitrogen use efficiency, trough stimulation of N-metabolism and accumulation of proteins, and hence to reduce the use of MF by 40%, thus decreasing the nitrate leaching. These findings suggest that the use of BPs as biostimulant greatly contributes to reduce the consumption of mineral fertilizers, and to mitigate the environmental impact caused by nutrients leaching, according to European common agricultural policy, that encourages R&D of new bioproducts for sustainable eco-friendly agriculture.

## Introduction

Nowadays, the bioeconomy concept requires to exploit sustainable renewable biomasses to produce of fuels, chemicals, and agrochemicals which human population needs. Researchers are trying to valorise biomasses from different sources as alternative feedstocks, focusing these objectives^1^. These latter objectives are quite difficult to reach as they are dependent on the availability of biomasses, and the economic aspects related to their collection. So far, most of the R&D work on the valorisation of biomass as renewable feedstock focused on processing plants and crops to be used for fuel production, raising social concerns due to the exploitation of agricultural land for the production of non-food energy crops. On the contrary, the use of biowastes as feedstock could mitigate the popular discomfort for the environmental impact of the increasing wastes production and current disposal practices.

Municipal biowaste (MBW) is the most available and sustainable potentially renewable feedstock. As two-thirds of world population is expected living in urban areas by 2050, and produce more wastes, the cities are crucial to the circular waste-based economy^2^. At present time, MBW is a social economic and environmental burden. Its valorization as feedstock producing valued added products would solve both problems. Currently, MBW is processed by anaerobic and aerobic fermentation, yielding biogas, anaerobic digestate and compost. The value of these products does not cover the processing costs. As collection and treatment costs are paid off by citizens’ taxes, MBW and its digestate and compost represent negative cost feedstocks^3^. Converting MBW, digestate and compost to value-added chemicals is potentially the way to improve current MBW treatment plants and turn them into eco-friendly biorefineries producing fuel and new multifunctional value-added biobased products (BPs) for use in the chemical industry, agriculture and waste treatment sectors^4^.

Recently, the performance of BPs in agriculture as plant growth biostimulants and antifungal agents had been reported^5^. The BPs, applied to the soil at 50–150 kg/ha, were demonstrated to be more sustainable and efficient plant biostimulants, in comparison to commercial mineral and organo-mineral products (e.g. leonardite), for the cultivation of several ornamental plant species, such as *Euphorbia x lomi* Rauh^6^, *Lantana camara*^7^, *Murraya paniculata*^8^, Hibiscus^9^, and vegetable species, such as tomato^10^, red pepper^11^, spinach^12^, maize^13^, bean^14^, oilseed rape^15^. These biobased products were reported also as potential enhancers of the seed germination process of cress, tomato, and lettuce at low concentrations ranging between 10 and 100 mgL^-1^^16^. On the other hand, they are also fungicides at 1000–5000 mgL^-1^ concentration against several pathogens as *Botrytis cinerea*, *Sclerotinia sclerotiorum*, *Monilia* sp., *Sclerotium rolfsii*, and *Phytophthora nicotianae*^16^.

Moving forward from the above findings, the present work addresses specifically the environmental issues arising from the current agricultural common practice to increase plant productivity using fertilizer doses higher than plant requirements. Exceeding fertilizer amounts accumulate in soil, could be leached into ground water, reach the food chain, and consequently may affect human and animal health^17^. Fertilizers are the leading cause for eutrophication, as they contain all the key ingredients for prosperous growth: nitrogen, phosphorous and potassium. Main fertilizers include inorganic NPK and organo-mineral products, such as composts of biowastes from urban, animal, agriculture sources, peat and leonardite hydrolysates^18^. Compared to nitrates, the most lixiviated nutrients from the soil, phosphates are only moderately soluble and not mobile in soils and groundwater. Phosphates tend to remain attached to soil particles, but erosion can transport considerable amounts of phosphate to streams and lakes. Depending on fertilizers’ dosage, soil type, and plant cultivated type, from 70 to 250 kg/ha nitrates leaching may occur. The Council Directive 91/676/EEC requires the reduction of water pollution caused or induced by nitrates from agricultural sources to prevent eutrophication processes^19^. To protect soil and waters from the negative environmental impact caused by fertilisers, while maintaining plant productivity and crop quality, the most recent EU Fertilizing Products Regulation effective from July 16, 2022 sets out minimum and maximum limits of C, N, K, P and heavy metals for fertilizers^20^.

The general goal of the present work was to investigate the effect of BPs on nitrogen metabolism in the plant-soil system, in order to evaluate further possible effects of these new products to reduce nutrient leaching in agriculture, while maintaining the plant productivity. As previous works suggested that the use of BPs may increase plant growth, the present work focuses on BPs effect on nitrogen adsorption and, consequently, on the reduction of nitrate lixiviation trough soil, thus contributing to reduce mineral fertilizers consumption, and to mitigate the environmental impact caused by leaching. To this end, in the present work a new species, lettuce, never tested before with BPs, was taken as case study. Growth parameters as well as plant biochemical response to the treatment were evaluated. The BPs effect on the plants was evaluated by monitoring the nitrogen flux in the plant, determining the activities of enzymes involved in the nitrogen uptake, such as nitrate reductase, glutamate synthase and glutamine synthase, as well as the nitrogen form accumulated in the plant tissues. Nitrate leaching during the cultivation of lettuce in pots was then evaluated.

## Materials and methods

### Materials

BPs were produced from the solid anaerobic digestate of MBW provided by the *ACEA Pinerolese Industriale S.p.A*. (Pinerolo, Turin, Italy) waste treatment plant^21^. In brief, the digestate was hydrolysed in water at pH 13 and 60 °C, then separated from the insoluble residue by sedimentation, followed by centrifugation and ultrafiltration. The membrane retentate was dried at 60 °C, and the solid product was dissolved in water at pH 10^16^. The obtained BPs was characterised for its chemical composition according to previous works^21,22^. Moreover, potentially toxic elements, Cu, Zn, Cd, Hg and Pb in obtained BPs were measured according to Padoan et al.^23^, by using microwave digestion (HNO_3_/H_2_O_2_ 4:1 v/v) on 1.0 g of sample (Milestone Ethos D). Pseudo-total contents were then quantified by inductively coupled plasma mass spectrometry (ICP-MS, PerkinElmer NexION® 350D). The accuracy was checked using a Reference Materials (NIST SRM 1572, National Institute of Standards and Technology, USA); all recoveries of analysed metals were between 90 and 110%. All measured heavy metals were lower than limit parameters determined by the Regulation (EU) 2019/1009, for Product Function Categories (PFC) 1 A) Solid Organic Fertiliser, PFC 1 B) Organic mineral Fertiliser, and PFC 6 B) Non microbial Plant Biostimulant (Table 1). Finally, the absence of pathogens in BPs is guaranteed by the high pH and temperature treatments subjected to.

**Table 1:** Heavy metal contents in BPs used in the experimental trials, and legal limit of Regulation (EU) 2019/1009 (PFC) 1 A): Solid Organic Fertiliser, PFC 1 B): Organic mineral Fertiliser, and PFC 6 B): Non microbial Plant Biostimulant).

Heavy metal	BPs (mg/kg d.m)	PFC 1 A) (mg/kg d.m)	PFC 1 B) (mg/kg d.m)	PFC 6 B) (mg/kg d.m)
Zn	256	800	1500	1500
Cu	202	300	600	600
Cd	< 0.5	1.5	3	3
Pb	85	120	120	120
Hg	0.2	1	1	1

Plant material, comply with relevant institutional, national, and international guidelines and legislation, and all methods were carried out in accordance with these relevant guidelines.

### Experimental conditions

The agriculture trials were performed in 1 kg soil pots (diameter 20 cm) in greenhouse conditions (27 August 2021 – 03 October 2021), in a farm located in Vittoria (Ragusa, Italy). Soil texture was evaluated using the pipette method, determining the particle size classes which were subdivided into clay, silt, and sand^24^. Particles > 2000 µm were not considered. The soil was air dried, sieved at 2 mm and characterized for water holding capacity (WHC), humidity, pH, electric conductivity (E.C.), organic carbon, phosphorus, total nitrogen, potassium, and Cation Exchange Capacity (C.E.C), following the procedures described in Puglisi et al.^25^. Soil characterization is reported in Table 2.

**Table 2:** Physical–chemical properties of the soil used in the experimental trials.

Clay (%)	Silt (%)	Sandy (%)	WHC (%)	Humidity (%)	pH	Electrical conductivity (mS/cm)	Organic carbon (%)	Total Nitrogen (g/kg)	P (mg/kg)	K (mg/kg)	C.E.C (cmols(+)/kg)
13.5	18.3	68.2	0.2	5.97	7.92	2.95	1.57	1.1	10	42	7.59

The soil was previously subjected to independent treatments, using two different dosage of BPs (50 kg/ha and 150 kg/ha), based on previous results obtained on other vegetable species^10–14^. The BPs were used alone or mixed with different amounts (100%, 60% and 0%) of mineral fertilizer (MF), and bured into the soil before transplant. The MF (solid ternary fertilizer NPK made of: NH_4_NO_3_, KH_2_PO_4_, and KNO_3_) used in the agriculture trials was purchased from a local agricultural supplier. Soils fertilized with MF only and non-fertilized were used as controls. MF 100% corresponds to the amounts used in the regular practice for lettuce cultivation 116.60 kg/ha NH_4_NO_3_, 162.32 kg/ha KH_2_PO_4_, and 138.60 kg/ha KNO_3_^26^, MF60% represents a 40% MF reduction with respect to the regular practice (69.96 kg/ha NH_4_NO_3_, 97.40 kg/ha KH_2_PO_4_, and 83.16 kg/ha KNO_3_), while MF0% means absence of mineral fertilization. Soil N, P, K contents in the different treatments were calculated based on the BPs and MF composition, and the amounts of nutrients supplied to the soil for each treatment are reported in Table 3.

**Table 3:** Nutrient amount of N, P, and K supplied to the soil with the treatments.

Treatment	N (kg/ha)	P (kg/ha)	K (kg/ha)
BPs150 + MF100%	66.01	37.34	105.37
BPs150 + MF60%	42.00	22.51	65.37
BPs150 + MF0%	6.01	0.34	5.37
BPs50 + MF100%	62.00	37.11	101.79
BPs50 + MF60%	38.00	22.31	61.79
BPs50 + MF0%	2.00	0.11	1.79
MF100%	60.00	37.00	100.00
MF60%	36.00	22.20	60.00
MF0%	–	–	–

Lettuce seedlings (*Lactuca sativa* var. romana), at four true leaves, were provided by a local nursery, and were transplanted (27 august 2021) in each pot in a completely randomized design composed by three replicas per treatment, and each replica was made of 10 seedlings. The seedlings were regularly grown in the soil treated as above described, and were irrigated every day, to maintain 50% WHC, by dripline sprinkler for 40^th^ days.

In order to simulate raining events naturally occurring, and hence possible phenomena of nitrate lixiviation into groundwater, two full supplemental irrigation treatments, consisting of an amount of water 1/3 greater than that needed to reach the WHC (280 ml), were performed after 8 and 28 days from the transplant. Then, the water lixiviated from pots, was collected and stored at − 80 °C until analyses.

At the end of the experimental period (40 days), lettuces were sampled, separated in root and shoot, and then the morphobiometric parameters were evaluated. The tissues were immediately frozen with liquid nitrogen and stored at − 80 °C until further use.

Soils were sampled, and immediately analysed for enzymatic activity. The remaining sample of soils were stored at − 20 °C until further analysis.

### Morphobiometric parameters of lettuce

Lettuce roots and shoots were separately weighed, in order to obtain the fresh weight of shoot (shoot FW) and root (root FW). Dry weight of lettuce tissues (shoot DW and root DW) was obtained by placing them in a drying oven at 105 °C until constant weight was reached, then allowed to cool for 2 h inside a closed bell jar, and finally the dry weights were calculated. Root lengths were measured with a flexible ruler to the nearest 0.5 mm.

All measurements were performed on 3 plants for treatment and replicates.

### Determination of the nitrogen forms in lettuce tissues

The nitrogen chlorophyll content of lettuce leaves, related to the nitrogen status of the plant, was measured, before the second over-irrigation event, using in field condition a portable N-Tester (Konica, Minolta, Japan), as average of three different points of the last expanded leaf of each lettuce plant, for all treatments and replicates^27^. The tool provides a numeric three-digit dimensionless value that is commonly expressed as N-Tester value, and is used for leaf chlorophyll estimation in lettuce^28^.

Total nitrogen was determined in leaves and roots by the Kjeldahl method, by digesting 2 g DW of tissues with concentrated sulphuric acid and selenium catalysis^29^.

Total protein extraction from lettuce tissues (root and leaf) was performed according to La Bella et al.^30^. Briefly, aliquots of lettuce leaves and roots were homogenized using an extraction buffer (1:1.25 w/v ratio) containing: 220 mM mannitol, 70 mM sucrose, 1 mM EGTA, 10 mM cysteine, and 5 mM HEPES–KOH pH 7.5. Samples were then filtered and centrifuged at 13,000 rpm for 30 min at 4 °C. The supernatant was recovered, and the total protein content was determined by the Bradford^31^ method, using Bovine Serum Albumine (BSA) as a standard curve, and expressed as mg protein g^-1^ DW. All measurements were performed on 3 plants for treatment and replicates.

Nitrate (N-NO_3_) concentration in leaves and roots, at the end of the trial, has been analysed on the fresh material. For each plant, 100 mg of fresh tissue was ground in liquid nitrogen and suspended in 10 mL of deionized water. Suspensions were incubated for 1 h at 45 °C and then centrifuged at 5,000 rpm for 15 min and filtered. The extract was used for nitrate spectrophotometric (U-2000, Hitachi, Tokyo, Japan) determination using the Griess reaction^32^.

### Enzymatic activities related to nitrogen metabolism in lettuce tissues

Each enzymatic activity was assayed using an aliquot of the total protein extract, obtained as previously described, containing crude enzyme extract.

Nitrate reductase (NR) activity was measured according to Kaiser et al.^33^ method. Briefly, a solution containing 100 mM KH_2_PO_4_ and 100 mM KNO_3_ was incubated at 28 °C for 15 min with the suitable amount of enzyme extract. The mixture was then centrifuged at 500 rpm, the supernatant was recovered, and the activity spectrophotometrically measured at 540 nm (Jasco V-530 UV–vis spectrophotometer, Tokyo, Japan), using a calibration curve, with known concentrations of NaNO_2_. Activity was expressed as Unit mg^-1^ protein.

Glutamine synthetase (GS) was performed according to Canovas et al.^34^. In brief, the assay mixture contained 90 mM imidazole–HCl (pH 7.0), 60 mM hydroxylamine (neutralized), 20 mM KAsO_4_, 3 mM MnCl_2_, 0.4 mM ADP, 120 mM glutamine, and the suitable amount of enzyme extract. The enzymatic reaction was incubated at 37 °C for 15 min, then a mixture (1:1:1) of 10% (w/v) FeCl_3_ 6H_2_O in 0.2 M HCl, 24% (w/v) trichloroacetic acid, and 50% (w/v) HCl was added. The activity was spectrophotometrically determined at 540 nm, using a standard curve of γ-glutamyl hydroxamate, and was expressed as µmol-glutamyl hydroxamate mg^-1^ protein min^-1^.

Glutamate synthase (GOGAT) activity was assayed as described by Avila et al.^35^. Briefly, the assay mixture, containing 25 mM Hepes–NaOH (pH 7.5), 2 mM L-glutamine, 1 mM.α-ketoglutaric acid, 0.1 mM NADH, 1 mM Na2EDTA, and the suitable amount of enzyme extract, was measured spectrophotometrically (Jasco V-530 UV–vis spectrophotometer, Tokyo, Japan), by following NADH oxidation at 340 nm. GOGAT activity was expressed as nmol NAD^+^ min^-1^, mg^-1^ protein, using a molar extinction coefficient of 6220 L mol^-1^ cm^-1^.

### Determination of the nitrogen forms in soil

The determination of nitrate nitrogen (NO_3_^−^N) was performed following the procedure described by Mulvaney^36^ and Miranda et al.^32^. Soil samples were air dried and sieved at 2 mm. Nitrogen forms were extracted from soil (10 g) with 1 M KCl, under mechanical agitation for 60 min and further centrifugation at 3000 rpm for 10 min. Nitrites were detected in the supernatants, by using Griess solution, which was prepared by mixing 0.1% naphthalene ethylenediamine hydrochloride (NED) and 1% sulfonamide in phosphoric acid. The reaction was developed at room temperature for 20 min, then was spectrophotometrically analysed at 540 nm, using a NO_2_^-^ standard curve. Nitrate was measured by its reduction to nitrite by vanadium(III), and calculating its concentration in the supernatants by subtracting the amount of nitrite previously determined. N-NO_3_ was expressed as mg N-form/g dry weight of soil (mg g^-1^ DW soil).

Total nitrogen was determined by the Kjeldahl method, by digesting 5 g of soil samples with concentrated sulphuric acid and selenium catalysis^37^.

### Determination of the N-NO_3_ in leached water

The nitrate content was determined in leached water after an extraction with 1 M KCl for 1 h, and then determined spectrophotometrically as above described for the soil, using Griess solution^32^.

### Nitrogen use efficiency parameters

Nitrogen uptake efficiency (NUpE), nitrogen utilization efficiency (NUtE), and nitrogen use efficiency (NUE) were calculated according to Xu et al.^38^.

In detail parameters were calculated as follows:

- Total N accumulation (TNA) = total N concentration × shoot DW (expressed as mg N);
- NUpE = TNA/root DW (expressed as mg N g^−1^ DW);
- NUtE = shoot DW/N concentration (expressed as g^2^ DW mg^−1^ N);
- NUE = NUtE × NUpE (g DW).

### Statistical analyses

Data were analysed by one-way ANOVA (*p* < 0.05) followed by Tukey’s test for multiple comparison procedures using the Statistics package software (version 10; Statsoft Inc., Tulsa, OK, USA) to investigate the effect of the treatment on plant, soil, and water analysis.

## Results

### Morphobiometric parameters of lettuce

The morphological traits of lettuce seedlings subjected to the BP treatments were measured, and the results are shown in Table 4. As expected, among controls, MF100% showed, for all the evaluated parameters, values greater than ones for MF60% and MF0% soil. The only exceptions were observed for root FW and DW, for which MF100% and MF60% registered similar values. At the shoot level, the best performances were obtained in the treatment BPs150 + MF100% and BPs150 + MF60%, recording for the FW of the edible portion, an increase of around 24% and 22% respect to the control MF100%, respectively. Moreover, also BPs50 + MF100% and BPs50 + MF60% showed significantly higher values than the control MF100% (e.g., shoot FW showed increases of 13% and 10%, respectively). Interestingly, the treatment BPs150 + MF0%, without added MF, recorded values always similar to MF100% in spite of the fact the applied nutrients were 1–2 order of magnitude lower. Similarly, at the root level, the highest values were obtained with the treatments BPs150 + MF100% and BPs150 + MF60%, recording a root FW 27% and 21% higher than the control MF100%, respectively, and a root length 17% and 19% higher than the control MF100%, respectively (Table 4). All other treatments showed parameters not significantly different from the control MF100%, except for root length, in which the treatment BPs50 + MF0% showed a value similar to the control MF60%, and lower than MF100%.

**Table 4:** Morphological traits of lettuce seedlings subjected to BP treatments.

Treatment	Shoot FW (g)	Shoot DW (g)	Root FW (g)	Root DW (g)	Root length (cm)
BPs150 + MF100%	79.05 ± 3.74 a	6.19 ± 0.31 a	24.10 ± 1.41 a	3.29 ± 0.13 a	17.07 ± 1.74 a
BPs150 + MF60%	77.56 ± 2.22 a	5.82 ± 0.19 a	22.97 ± 2.19 a	3.19 ± 0.30 a	17.43 ± 0.80 a
BPs150 + MF0%	65.53 ± 1.16 c	4.84 ± 0.23 c	19.37 ± 0.86 b	2.91 ± 0.12 b	15.13 ± 2.40 b
BPs50 + MF100%	72.25 ± 5.24 b	5.35 ± 0.46 b	18.27 ± 2.75 b	2.67 ± 0.51 b	15.13 ± 0.94 b
BPs50 + MF60%	69.75 ± 4.24 b	5.09 ± 0.33 b	18.47 ± 1.33 b	2.72 ± 0.14 b	15.07 ± 1.85 b
BPs50 + MF0%	55.99 ± 3.75 e	3.76 ± 0.45 e	18.66 ± 0.97 b	2.75 ± 0.14 b	14.23 ± 0.29 c
MF100%	63.78 ± 2.47 c	4.81 ± 0.49 c	19.00 ± 1.26 b	2.55 ± 0.17 b	14.57 ± 1.82 b
MF60%	59.09 ± 1.48 d	4.23 ± 0.34 d	17.71 ± 1.18 b	2.16 ± 0.25 b	13.97 ± 0.47 c
MF0%	45.33 ± 2.47 f	2.81 ± 0.36 f	14.63 ± 0.88 c	1.75 ± 0.19 c	11.14 ± 1.63 d

Data are means ± SD. Values in the same column followed by different letters are significantly different (*p* < 0.05).

### Nitrogen forms in lettuce tissues

**Figure 1:**
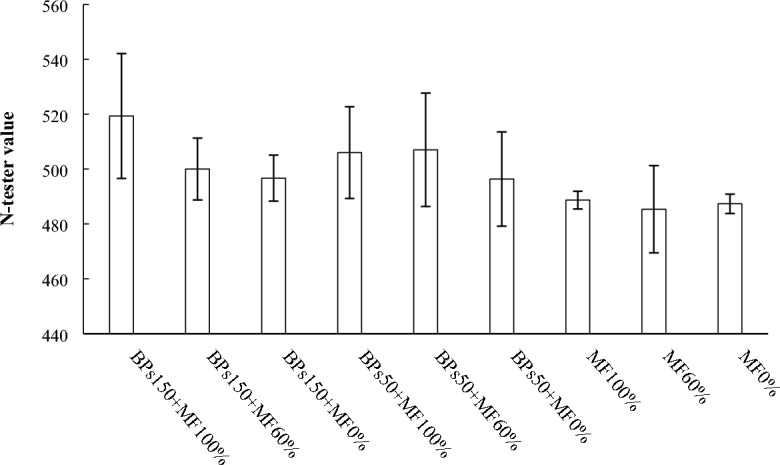
Nitrogen status of the plant during cultivation, before the second over-irrigation event. Values are reported as indices described by N-Tester. Error bars indicate standard deviation ± SD. The absence of letters above the columns shows the lack of significant differences.

The nitrogen status of the plant was monitored in field using a N-Tester, prior to the second over-irrigation event, as described in the Material and Methods section. The values (Fig. 1) showed that no significant differences were recorded among treatments.

**Figure 2:**
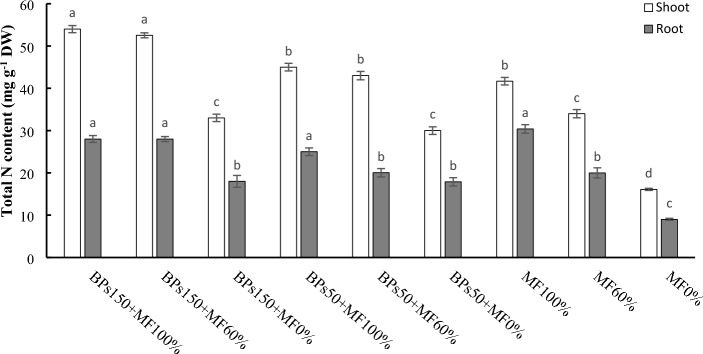
Total nitrogen (N) content in lettuce tissues (shoot and root). Error bars indicate standard deviation ± SD. Values followed by different letters are significantly different (*p* < 0.05).

The total N content in leaves (Fig. 2) significantly increased in the treatments with BPs150 + MF100% and BPs150 + MF60%, respect to the control with MF 100% (29% and 26%, respectively). The treatments BPs50 + MF100% and BPs50 + MF60% showed total N values similar to the control MF100%, thus indicating a potential fertilization saving of 40%. The treatments with BPs50 + MF0% and BPs150 + MF0%, showed an amount of total nitrogen lower than MF100%, but not significantly different from the control MF60%. At the root level, the treatments BPs150 + MF100%, BPs150 + MF60%, and BPs50 + MF100% showed N values similar to the control MF100%, whereas treatments BPs50 + MF60%, BPs50 + MF0%, and BPs150 + MF0% recorded values lower than control MF100%, but not significantly different from the control MF60%.

**Figure 3:**
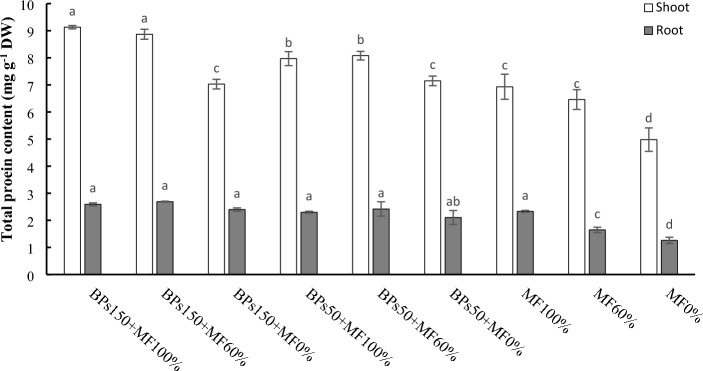
Total protein content in lettuce tissues (shoot and root). Error bars indicate standard deviation ± SD. Values followed by different letters are significantly different (*p* < 0.05).

Figure 3 reports the content of the total proteins extracted from lettuce tissues. The total protein content in leaves was strongly influenced by the treatments, recording a significantly increase in BPs150 + MF100% and BPs150 + MF60%, respect to the control with MF100% (32% and 28%, respectively). The treatments BPs50 + MF100% and BPs50 + MF60% also raised the protein content of the lettuce epigeal part, as compared to the control MF 100% (around 16%). Finally, in both the treatments with the two BPs dosage without mineral fertilizations (BPs150 + MF0% and BPs50 + MF0%), values always similar to MF100% and MF60% occurred. As previously reported for N total, a fertilization reduced of 40% leads to similar protein content as with the regular fertilization. At the root level, all the treatments showed not significant differences respect to the control MF100%, although they showed values higher than MF60%.

**Figure 4:**
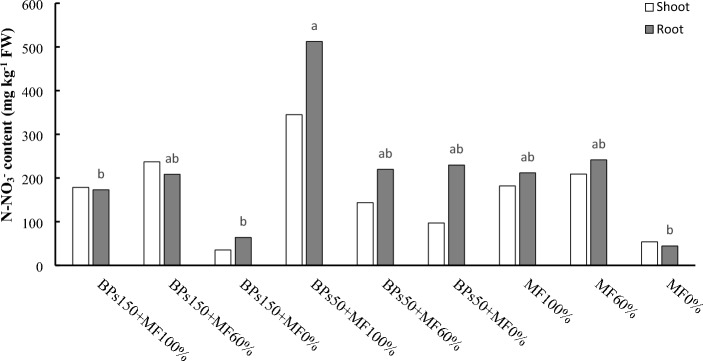
N-NO_3_^−^ content content in lettuce tissues (shoot and root). Values followed by different letters are significantly different (*p* < 0.05). The absence of letters above the columns shows the lack of significant differences.

The N-NO_3_^−^ content extracted from lettuce tissues is reported in Fig. 4. In leaves, due to the great variability of N-NO_3_^−^ values in the replicates, no significant differences were observed among treatments. In roots a great variability of N-NO_3_^−^ also occurred. However, in both cases, the highest value was recorded for BPs50 + MF100%. This value, although not significantly higher than those for most of the other treatments, was significantly higher than the lowest value recorded for the treatments BPs150 + MF100%, and BPs150 + MF0% and MF0%.

### Enzymatic activities related to the nitrogen metabolism in lettuce tissues

**Figure 5:**
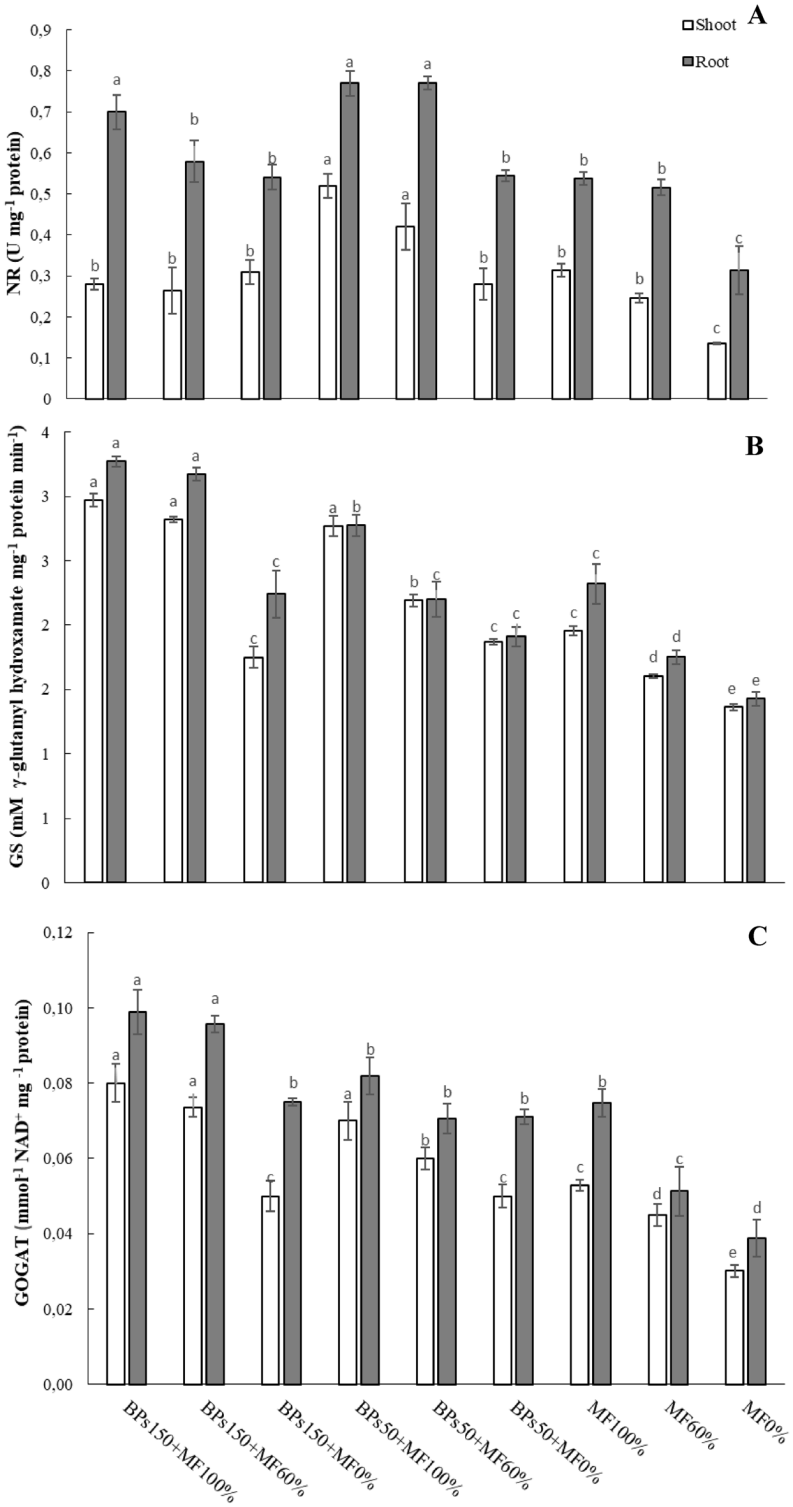
Nitrate reductase (NR) activity (**A**), glutamine synthase (GS) activity (**B**), glutamate synthase (GOGAT) activity (**C**) in lettuce tissues (shoot and root). Error bars indicate standard deviation ± SD. Values followed by different letters are significantly different (*p* < 0.05).

Figure 5 reports the enzymatic activities of nitrate reductase (NR), glutamine synthetase (GS), and glutamate synthase (GOGAT) in the lettuce plants grown on soil subjected to the treatments listed in Table 2.

NR activity, measured in lettuce leaves (Fig. 5A), increased respect to the control MF100% by about 68% under the treatment BPs50 + MF100%, and around 35% under the treatment BPs50 + MF60%. All other treatments showed NR activity values in the shoot similar to the control MF100%. In roots, the treatments BPs50 + MF100%, BPs150 + MF100%, and BPs50 + MF60% rapidly induced the activation of GS, reaching values of activity 43%, 30%, and 44%, respectively, higher than that measured in the control MF100%.

GS activity in leaves was significantly higher in the plants treated with BPs150 + MF100% (52%), BPs150 + MF60% (44%), and BPs50 + MF100% (41%) respect to the control MF100%, followed by BPs50 + MF60% (12% higher than MF 100%), whereas all other treatments showed values of activity always similar to the control. As regard roots, the highest values of activity were recorded in the treatments BPs150 + MF100% (41% higher than MF100%) and BPs150 + MF60% (37% higher than MF100%). The treatment BPs50 + MF100% showed an activity lower than these latter, but higher than the MF100%. All other treatments showed activities similar to the control (Fig. 5B).

GOGAT activity in leaves showed a trend very similar to GS activity, recording the highest values under the treatments with BPs150 + MF100% (57%), BPs150 + MF60% (47%), and BPs50 + MF100% (42%), respect to the control MF 100%. The treatment BPs50 + MF60% showed an activity 25% higher than MF100%, whereas the treatments without MF (BPs150 + MF0% and BPs50 + MF0%) showed values of activity not significantly different from the control. As regard roots, all the treatments showed values of activity similar to the control MF100%, except the treatments BPs150 + MF100% and BPs150 + MF60%, which showed an increase respect to the control of 32% and 28%, respectively (Fig. 5C).

### N-NO_3_^−^ in soil

**Figure 6:**
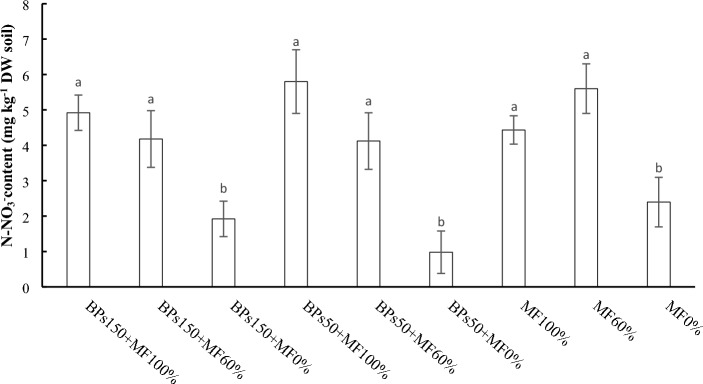
N-NO_3_^−^ content in the soil at the end of the experimental trials. Error bars indicate standard deviation ± SD. Values followed by different letters are significantly different (*p* < 0.05).

**Figure 7:**
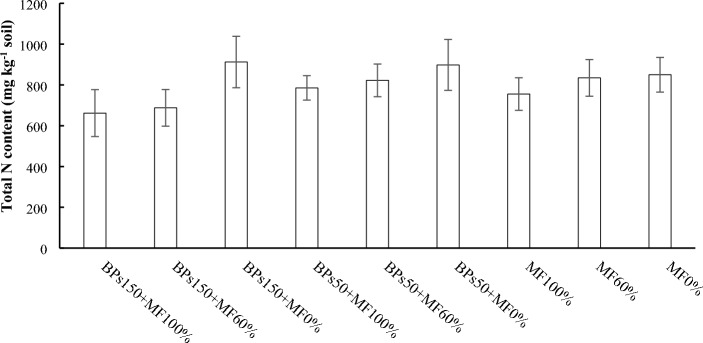
Total nitrogen (N) content in the soil at the end of the experimental trials. Error bars indicate standard deviation ± SD. The absence of letters above the columns shows the lack of significant differences.

Figures 6 and 7 report the N-NO_3_^−^ and total N measured in soil at the end of the experimental trials. The N-NO_3_^−^ data showed no significant differences between soils treated with the mineral fertilisers only (MF100% and MF60%) or with the MF-BS mixes. All the treatments with MF gave higher N-NO_3_^−^ values than the values measured for the control MF0%. The treatments with BS only (BS150-MF0% and BS-MF0%) resulted not significantly different from MF0%. On the contrary, the soils treated with BPs exhibited the highest total N values, although these resulted not significantly different from values measured for all other treatments.

The total N content in soils, at the end of the experimental trials, showed that not significant differences among treatments and controls MF60% and MF0% occurred (Fig. 7).

### N-NO_3_^−^ content in leached water

**Figure 8:**
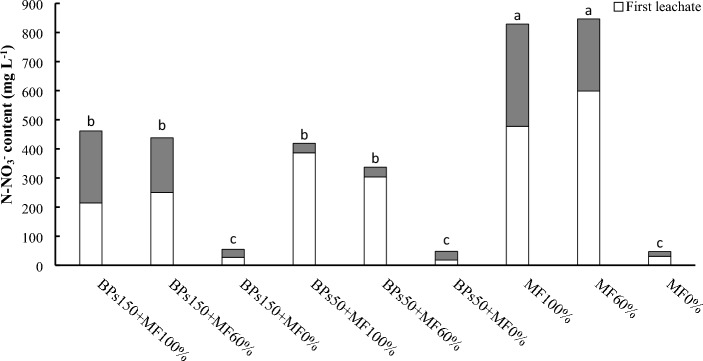
Nitrogen form N-NO_3_ in the water leached (First and Second leachate) during the experimental trials. Values followed by different letters are significantly different (*p* < 0.05).

Figure 8 reports the N-NO_3_^−^ contents in waters leached during the experimental trials. The data evidenced three groups of values significantly different one from the other. The MF100% and MF60% group showed the highest total average value (838 mg L^-1^). The second group, including the treatments with the BS-MF mixes, showed the highest total average value (471 mg L^-1^). The third group, including the BS150% + MF0%, BS50% + MF0% and MF0% treatments, showed the lowest total average value of 50 mg L^-1^. In terms of reduction of N-NO_3_^−^ leaching relatively to the first group, the second and third group exhibit reduction of 44% and 94%, respectively.

### Nitrogen efficiency parameters

Table 5 reports the values of the Nitrogen efficiency parameters measured for the different soil treatments. The plants grown in fertilized soils with the BPs-MF mixtures showed the highest TNA, NUpE and NUE values, always higher than all other treatments. The BPs150 + MF100% treatment exhibited the highest values. The NUE for this treatment showed an increase of 28% respect to the MF 100% treatment, and 158% respect to the control MF0%.

**Table 5:** Nitrogen efficiency parameters in lettuce seedlings subjected to BP treatments.

Treatment	TNA (mg N)	NUpE (mg N g^-1^DW)	NUtE (g^2^ DW mg^-1^N)	NUE (g DW)
BPs150 + MF100%	334.26 ± 12.01a	101.60 ± 2.11a	0.11 ± 0.04a	11.65 ± 0.52a
BPs150 + MF60%	305.75 ± 15.72b	95.85 ± 3.42b	0.11 ± 0.03a	10.62 ± 0.39b
BPs150 + MF0%	159.72 ± 11.32e	54.89 ± 5.65e	0.15 ± 0.04a	8.05 ± 0.21d
BPs50 + MF100%	240.75 ± 10.03c	90.17 ± 3.70b	0.12 ± 0.02a	10.72 ± 0.20b
BPs50 + MF60%	218.87 ± 8.50d	80.47 ± 2.25c	0.12 ± 0.01a	9.53 ± 0.42c
BPs50 + MF0%	112.8 ± 9.68f.	41.02 ± 8,98e	0.13 ± 0.01a	5.14 ± 0.53e
MF100%	200.46 ± 11.23d	78.61 ± 3.21c	0.12 ± 0.01a	9.07 ± 0.21c
MF60%	143.82 ± 10.21e	66.58 ± 2.87d	0.12 ± 0.02a	8.28 ± 0.12d
MF0%	45.22 ± 15.39g	25.84 ± 4.56f	0.17 ± 0.05a	4.51 ± 0.25e

Values in the same column followed by different letters are significantly different (*p* < 0.05).

## Discussion

Several studies evaluated the biostimulant effect of BPs on a wide range of crops^5^, but BPs for lettuce cultivation had never been tested. In lettuce at the shoot level, the treatments BPs150 + MF100% (+ 24% respect to MF100%) and BPs150 + MF60% (+ 22% respect to MF100%) determined a relevant increase of the FW of the edible portion, in accordance with the biostimulant effects observed for other species^10–15^. Moreover, the treatment with the highest amount of BPs without fertilization (BPs150 + MF0%) showed values of FW of the edible part comparable to MF100%, suggesting that BPs may be useful to ameliorate the use of the residual nutrients into the soil. The highest amount of BPs (150 kg/ha), both with MF100% or MF60%, determined also a positive effect at the root level, recording higher FW values in the treatments BPs150 + MF100% and BPs150 + MF60%, respect to the control (Table 4). Starting from the positive effect on the morphobiometric traits of the lettuce seedlings, the fate of the nitrogen (N) was investigated, as N represents the most important macronutrient in lettuce production for proper foliage growth and good green colour^39^. During lettuce cultivation, nitrogen status of the plant was monitored in field, using a non-invasive technique, and the results showed a great variability in the measurements with values not significantly different among the treatments (Fig. 1). N-test readings have been proven to be well correlated with the leaf chlorophyll content and/or leaf N concentration in several cereals such as *Hordeum vulgare* L.^40^, *Zea mays* L^41^, *Oryza sativa* L.^42^, and wheat^43^. These evidences suggest that, during the experimental trials, chlorophyll content keeps rather constant values. Moreover, according to Pennisi et al.^28^, who reported values of N-tester for lettuce ranging between 300 and 400, lettuce treated with 150 kg/ha BPs reached values ranging between 500–520, thus suggesting the presence of a great amount of chlorophyll in their leaves.

On the contrary, significant differences were observed as regard the different forms of nitrogen accumulated in lettuce tissues at the end of the experimental period. The treatments BPs150 + MF100% and BPs150 + MF60% greatly affected the accumulation of total nitrogen (N) and proteins at the shoot level of the lettuce (Fig. 2 and 3). This increased protein content is compatible in order to support the enhanced growth of the epigeous part of lettuce^44^. However, the increased N absorption efficiency of the plant, on the other hand, may lead to nitrate accumulation^45^. Lettuce leaves can accumulate a wide range of nitrate, varying from 190 to 6600 mg kg^−1^, depending on different factor such as species, individual plant, cultivation season, age, morphotype, climate, and fertilisation^46^. Risks related to high levels of nitrate are mainly related to methemoglobinemia, a disease affecting infants leading to anoxia or death, toxicity due to carcinogenic and mutagenic nitrosamine compounds, and associated to gastric cancer, due to the ingestion of N-nitroso compounds^47,48^. Moreover, a high nitrate levels in the edible part of baby leaf lettuce may determine a decrease of vitamins and hence of the nutritional profile^49^. Therefore, research is focusing on the use of techniques or treatments increasing N absorptions, but reducing its accumulation under form of nitrate. In Italy the presence of nitrate in lettuce is regulated by EU regulation N. 1258/2011, taking into account EFSA opinions^50,51^, indicating for lettuce cultivated in greenhouse a limit of nitrate corresponding to 4000 mg kg^-1^, between 1 April—30 September, and 5000 mg kg^-1^, between 1 October—30 March. Successfully, our results suggest that both the treatments 150BPs + MF100% and 150BPs + MF60% raised the total N accumulation in lettuce leaves (Fig. 2), by increasing the total protein content (Fig. 3), and nevertheless maintaining the levels of nitrate (Fig. 4) similar to those of control plants (MF100%, MF60% and MF0%). Interestingly, the highest value of nitrate, observed in BPs50 + MF100%, showed anyway a value (320 mg kg^-1^ FW) greatly lower than legal limits (4000–5000 mg kg^-1^).

In plants, nitrate may be metabolized both in shoots and roots, and the rate of its conversion is dependent on different environmental factors, type and amount of N supply, plant species, and age^52^. Nitrate reductase (NR) is a cytosolic enzyme that may be considered as the rate-limiting stage of the nitrate assimilation pathway, and it is considered to be a limiting factor for the growth and development of plants. NR, in the cytosol of plant cells, catalyses the reduction of NO_3_^−^ into NO_2_^−^, and acts as a crucial point in the plant N metabolism^53^. Our results showed that, in the soil with MF100%, the treatments with both concentration of BPs, significantly increased NR activities in roots, whereas in leaves NR activities were higher in the treatments with the lower amount of BPs (BPs50 + MF100% and BPs50 + MF60%) (Fig. 5A). These results may be explained by the evidence that higher N accumulation in lettuce correspond to a higher NR activity during the initial stage of plant growth, whereas a decrease of NR activity during the final stage of plant growth may occur^54^.

In the primary metabolism involved in N assimilation, the glutamine synthetase (GS) and glutamate synthase (GOGAT) have also been proposed to play a key role through ammonium incorporation into carbon skeletons, by assimilating the cation into an organic form as glutamine and glutamate^55,56^. Both GS and GOGAT, significantly increased in treatments BPs150 + MF100% and BPs150 + MF60% (Fig. 5B,C), in accordance with an increased growth of lettuce, and a higher amount of total N and proteins. Supporting these results, the involvement of N metabolism in the enhanced growth of lettuce was also observed using other biostimulant types, such as microalgae-based extracts^27,30,57^, plant-based preparations containing triacontanol^58^, l-amino acid-based biostimulants^59^.

It is well known that nitrogen is distributed into the plant, in the fixed fraction into the soil, and in the leached water^39^. Our results showed that, although the N total of all the soils was quite similar (Fig. 7), significant differences were observed as regard nitrate concentrations (Fig. 6). Interestingly, all the soils subjected to fertilization, both MF100% and MF60%, showed amount of NO_3_^−^ rather similar among them, and always greater than soils not fertilized (BPs150 + MF0%, BPs50 + MF0%, and MF0%). Meanwhile, NO_3_^−^ amounts in leachates significantly decreased in all waters collected from the fertilized soils (both 100% and 60%) subjected to BP treatments (both concentrations) (Fig. 8). All these results taken together, suggest that nitrate keeps constant in the fertilized soils for two different reasons: i) in the control fertilized soils (MF100% and MF 60%), the residual amount of nitrate (Fig. 6), after the plant uptake, may be strictly linked to the loss of NO_3_^−^ by lixiviation (Fig. 8); ii) on the contrary, the plants grown in fertilized soils and treated with BPs (BPs150 + MF100%, BPs50 + MF100%, BPs150 + MF60%, and BPs50 + MF60%) seem to uptake an higher amount of NO_3_^−^ from the soil, in order to support a greater growth of lettuce (Table 4), by increasing total protein content in the edible portion, and hence greatly reducing the amount of leached nitrate in the waters (Fig. 8). This hypothesis is supported by the evidence that, among the mechanisms of action of biostimulants based on humic-like substances, the increased uptake of nutrients such as nitrogen from the soil is one of the main studied processes^60–62^.

In this context, the nitrogen use efficiency (NUE) is considered a further important parameter, being related to the produced biomass per unit of available N. This parameter takes into account two factors: N uptake efficiency (NUpE), representing the ability of the plant to absorb N from the soil, and N utilization efficiency (NUtE), representing the potentiality of the plant to transfer and utilize N in the biomass production of the different plant tissues^38^. Our results showed that BPs, in particular BPs150 + M100%, increased the NUE respect to the lettuce grown in MF100% (Table 4). According to Lemaire et al.^63^, higher NUE improves the yield and quality of the plant, and decreases the environmental impact caused by the lixiviation of excess N fertilizer application. Moreover, in lettuce cultivation, Navarro-Leòn et al.^59^, have recently shown that the use of L-amino acid-based biostimulants improves nitrogen use efficiency (NUE), associated to NO_3_^−^ and total N accumulation in the plants. Our results hence suggest that 150 kg/ha BPs may be possible candidates to increase the lettuce growth, trough stimulation of N-metabolism, to reduce mineral fertilization, as the treatment BPs150 + MF60% showed results very similar to the treatment BPs150 + MF100%, and finally to decrease the nitrate concentration into groundwater.

Table 6 shows summarises the effects of the different treatments on the measured parameters.

**Table 6:** Ranking of the different treatments in the order of decreasing effects on the measured parameters.

Plant performance indicator	Ranking order	Δ1%^a^
Shoot FW (Table 4)	BPs150 + MF100% = BPs150 + MF60% > BPs50 + MF100% = BPs50 + MF60% > BPs150 + MF0% = MF100% > MF60% > BPs50 + MF0% > MF0%	74
Shoot DW (Table 4)	BPs150 + MF100% = BPs150 + MF60% > BPs50 + MF100% = BPs50 + MF60% > BPs150 + MF0% = MF100% > MF60% > BPs50 + MF0% > MF0%	120
Root FW (Table 4)	BPs150 + MF100% = BPs150 + MF60% > BPs50 + MF100% = BPs50 + MF60% = BPs150 + MF0% = MF100% = MF60% = BPs50 + MF0% > MF0%	64.7
Root DW (Table 4)	BPs150 + MF100% = BPs150 + MF60% > BPs50 + MF100% = BPs50 + MF60% = BPs150 + MF0% = MF100% = MF60% = BPs50 + MF0% > MF0%	88
Root length (Table 4)	BPs150 + MF100% = BPs150 + MF60% > BPs50 + MF100% = BPs50 + MF60% = BPs150 + MF0% = MF100% > MF60% = BPs50 + MF0% > MF0%	53
Leaves N-test (Fig. 1)	BPs150 + MF100% = BPs150 + MF60% = BPs50 + MF100% = BPs50 + MF60% = BPs150 + MF0% = MF100% = MF60% = BPs50 + MF0% = MF0%	6.6 ns
Leaves Total N (Fig. 2)	BPs150 + MF100% = BPs150 + MF60% > BPs50 + MF100% = BPs50 + MF60% = MF100% > BPs150 + MF0% = MF60% = BPs50 + MF0% > MF0%	223
Roots Total N (Fig. 2)	MF100% = BPs150 + MF100% = BPs150 + MF60% = BPs50 + MF100% > BPs50 + MF60% = BPs150 + MF0% = BPs50 + MF0% = MF60% > MF0%q	250
Leaf total proteins (Fig. 3)	BPs150 + MF100% = BPs150 + MF60% > BPs50 + MF100% = BPs50 + MF60% > BPs150 + MF0% = BPs50 + MF0% = MF60% = MF100% = MF60% > MF0%	67
Root total proteins (Fig. 3)	BPs150 + MF100% = BPs150 + MF60% = BPs50 + MF100% = BPs50 + MF60% = BPs150 + MF0% = BPs50 + MF0% = MF100% > MF60% > MF0%	75
Roots N-NO_3_ (Fig. 4)	BPs50 + MF100% ≥ BPs150 + MF60% = BPs50 + MF60% = BPs50 + MF0% = MF100% = MF60% > BPs150 + MF100% = BPs150 + MF0% = MF0%	90
NR activity in leaves (Fig. 5A)	BPs50 + MF100% = BPs50 + MF60% > BPs150 + MF60% = BPs50 + MF0% = MF100% = MF60% = BPs150 + MF100% = BPs150 + MF0% > MF0%	200
NR activity in roots (Fig. 5A)	BPs50 + MF100% = BPs150 + MF60% = BPs150 + MF100% > BPs50 + MF60% = BPs50 + MF0% = MF100% = MF60% = BPs150 + MF0% > MF0%	120
GS activity in leaves (Fig. 5B)	BPs150 + MF100% = BPs50 + MF100% = BPs150 + MF60% > BPs50 + MF60% > BPs50 + MF0% = MF100% = BPs150 + MF0% > MF60% > MF0%	50
GS activity in roots (Fig. 5B)	BPs150 + MF100% = BPs150 + MF60% > BPs50 + MF100% > BPs50 + MF60% = BPs50 + MF0% = MF100% = BPs150 + MF0% > MF60% > MF0%	125
GOGAT activity in leaves (Fig. 5C)	BPs150 + MF100% = BPs50 + MF100% = BPs150 + MF60% > BPs50 + MF60% > BPs50 + MF0% = MF100% = BPs150 + MF0% > MF60% > MF0%	167
GOGAT activity in roots (Fig. 5C)	BPs150 + MF100% = BPs50 + MF100% > BPs150 + MF60% = BPs50 + MF60% = BPs50 + MF0% = MF100% = BPs150 + MF0% > MF60% > MF0%	186
Soil N-NO_3_^−^ (Fig. 6)	BPs50 + MF100% = MF60% = BPs150 + MF100% = BPs150 + MF60% = BPs50 + MF60% = MF100% > MF0% = BPs150 + MF0% = BPs50 + MF0%	510
Soil total N (Fig. 7)	BPs150 + MF0% = BPs50 + MF0% = MF60% = BPs150 + MF100% = BPs150 + MF60% = BPs50 + MF60% = MF100% = MF0% = BPs50 + MF100%	32 ns
N-NO_3_^−^ in leached water (Fig. 8)	MF100% = MF60% > BPs150 + MF100% = BPs150 + MF60% = BPs50 + MF100% = BPs50 + MF60% > BPs150 + MF0% = BPs50 + MF0% = MF0%	1575
TNA (Table 5)	BPs150 + MF100% > BPs150 + MF60% > BPs50 + MF100% > BPs50 + MF60% = MF100% > BPs150 + MF0% = MF60% > BPs50 + MF0% > MF0%	642
NUpE (Table 5)	BPs150 + MF100% > BPs150 + MF60% = BPs50 + MF100% > BPs50 + MF60% = MF100% > MF60% > BPs150 + MF0% = BPs50 + MF0% > MF0%	293
NUtE (Table 5)	BPs150 + MF100% = BPs150 + MF60% = BPs50 + MF100% = BPs50 + MF60% = MF100% = MF60% = BPs150 + MF0% = BPs50 + MF0% = MF0%	54 ns
NUE (Table 5)	BPs150 + MF100% > BPs50 + MF100% = BPs150 + MF60% > BPs50 + MF60% = MF100% > BPs150 + MF0% = MF60% > BPs50 + MF0% = MF0%	158

^a^% increase of first ranking (or first listed), relatively to last ranking (or last listed) calculated according to 100 (first–last)/last ranking values; ns = not significant.

It may be readily observed that, for all measured parameters, the treatments with the BPs-MF mixes rank first and exhibit the highest effects, compared to the treatments with MF only or BPs only, and with the control MF0%. Particularly significant is the N-NO_3_^−^ in leached water 1575% increase measured for the treatment with MF100% and MF60%, relatively to the BPs150 + MF0% and BPs50 + MF0%, which together with the control MF0% trial exhibited the lowest N-NO_3_^−^ value in leached water. This prospects that formulates containing both MF and BPs in the proper relative amounts can achieve the highest plant productivity, together with the lowest environmental impact from fertiliser leaching in waters through the soil and the best safest crop quality.

With reference to the goal of lowering the consumption of mineral fertilizers, and the consequent depletion of mineral fossil sources, production on energy intensive N compounds and related GHG production, and finally the European import of mineral fertilisers, by implementation of BPs as alternative/supplementation to commercial MF, Table 3 shows that, compared to the MF100% and MF60% treatments, the use of BPs150 + MF0% and BPs50 + MF0 implies a strong reduction of mineral fertilisers supplied. Generally, according to Table 3 data, the use of all BPs-MF mixes, except for BPs150 + MF100%, would result in a reduction of N, P, K amounts.

## Conclusions

Considering all the concerns associated with nitrogen fertilization, nowadays it is essential to use new agronomic techniques able to increase NUE by plants and reduce the environmental impact linked to the lixiviation of nitrogen. In this context, the use of biostimulants has the potentiality to address some of the problems related to N fertilization. The present work has shown new evidences about BPs biostimulant properties on lettuce, a new species never tested before with BPs. Our results showed that 150 kg/ha BPs are able to increase lettuce growth, enhance NUE, and in the meantime reduce the loss of N thought lixiviation. In particular, the use of BPs in lettuce cultivation has shown to increase its growth, improve the nitrogen adsorption, thought the stimulation of N metabolism and the protein accumulation, allowing to reduce of 40% the consumption of mineral fertilizers. Moreover, BPs by increasing the N uptake are also effective to reduce the nitrate lixiviation trough the soil, thus contributing to mitigate the environmental impact caused by leaching.

The results of this paper lead the basis for a further sustainable exploitation of biowaste materials, thus contributing to a more circular economy, which allows to better address the nitrogen fate, prospecting a feasible development of new BPs-based farming practices for a more sustainable agriculture.

## Data Availability

All data generated during this study are included in this published article. The datasets analysed during the current study are available from the corresponding author on reasonable request.

## References

[1] Sharew S, Montastruc L, Yimam A, Negny S, Ferrasse JH (2022). Alternative energy potential and conversion efficiency of biomass into target biofuels: A case study in ethiopian sugar industry- Wonji-Shoa. Biomass.

[2] Paiho S (2021). Creating a circular city—An analysis of potential transportation, energy and food solutions in a case district. SCS.

[3] Montoneri E (2022). Integrated chemical and biochemical technology to produce biogas with a reduced ammonia content from municipal biowaste. Validating lab-scale research in a real operational environment. Environ. Sci. Adv..

[4] Montoneri E, Morone P, Papendiek F, Tatiu VE (2017). Food waste reduction and valorisation: Sustainability assessment and policy analysis. Municipal Waste Treatment, Technological Scale Up and Commercial Exploitation: The Case of Bio-Waste Lignin to Soluble Lignin-Like Polymers.

[5] Montoneri E, Baglieri A, Fascella G (2022). Biostimulant effects of waste derived biobased products in the cultivation of ornamental and food plants. Agriculture.

[6] Fascella G, Montoneri E, Ginepro M, Francavilla M (2015). Effect of urban biowaste derived soluble substances on growth, photosynthesis and ornamental value of *Euphorbia x lomi*. Sci. Hortic..

[7] Fascella G, Montoneri E, Francavilla M (2018). Biowaste versus fossil sourced auxiliaries for plant cultivation: The Lantana case study. J. Clean. Prod..

[8] Fascella G, Montoneri E, Rouphael Y (2021). Biowaste-derived humic-like substances improve growth and quality of orange Jasmine (*Murraya paniculata* L. Jacq.) plants in soilless potted culture. Resources.

[9] Massa D (2016). Application of municipal biowaste derived products in Hibiscus cultivation: Effect on leaf gaseous exchange activity, and plant biomass accumulation and quality. Sci. Hortic..

[10] Sortino O (2012). Refuse derived soluble bio-organics enhancing tomato plant growth and productivity. Waste Manag..

[11] Sortino O, Dipasquale M, Montoneri E, Tomasso L, Avetta P, Bianco Prevot A (2013). 90% yield increase of red pepper with unexpectedly low doses of compost soluble substances. Agron. Sustain. Dev..

[12] Padoan E (2022). Waste biopolymers for eco-friendly agriculture and safe food production. Coatings.

[13] Rovero A (2015). Sustainable maize production by urban biowaste products. Int. J. Agron. Agric. Res..

[14] Baglieri A (2014). Fertilization of bean plants with tomato plants hydrolysates. Effect on biomass production, chlorophyll content and N assimilation. Sci. Hortic..

[15] Jindrichova B, Burketova L, Montoneri E, Francavilla M (2018). Biowaste-derived hydrolysates as plant disease suppressants for oilseed rape. J. Clean. Prod..

[16] Fragalà F (2022). New insights into municipal biowaste derived products as promoters of seed germination and potential antifungal compounds for sustainable agriculture. Chem. Biol. Technol. Agric..

[17] Vanni A, Anfossi L, Cignetti A, Baglieri A, Gennari M (2006). Degradation of pyrimethanil in soil: Influence of light, oxygen, and microbial activity. J. Environ. Sci. Health B.

[18] Barone V (2019). Molecular and morphological changes induced by leonardite-based biostimulant in *Beta vulgaris* L. Plants.

[19] Council Directive 91/676/EEC of 12 December 1991 concerning the protection of waters against pollution caused by nitrates from agricultural sources. https://eur-lex.europa.eu/IT/legal-content/summary/fighting-water-pollution-from-agricultural-nitrates.html (2023).

[20] European Commission. Regulation (EU) 2019/1009—The Fertilising Products Regulation. https://eur-lex.europa.eu/eli/reg/2019/1009/oj (2023).

[21] Montoneri E (2020). High molecular weight biosurfactants from mild chemical reactions of fermented municipal biowastes. ChemistrySelect.

[22] Rosso D, Fan J, Montoneri E, Negre M, Clark J, Mainero D (2015). Conventional and microwave assisted hydrolysis of urban biowastes to added value lignin-like products. Green Chem..

[23] Padoan E, Passarella I, Prati M, Bergante S, Facciotto G, Ajmone-Marsan F (2020). The suitability of short rotation coppice crops for phytoremediation of urban soils. Appl. Sci..

[24] Violante P (2000). Metodi di analisi chimica del suolo.

[25] Puglisi I (2019). Physiological and biochemical responses of orange trees to different deficit irrigation regimes. Plants.

[26] Muscolo A, Marra F, Canino F, Maffia A, Mallamaci C, Russo M (2022). Growth, nutritional quality and antioxidant capacity of lettuce grown on two different soils with sulphur-based fertilizer, organic and chemical fertilizers. Sci. Hortic..

[27] Puglisi I, La Bella E, Rovetto EI, Stevanato P, Fascella G, Baglieri A (2022). Morpho-biometric and biochemical responses in lettuce seedlings treated by different application methods of *Chlorella vulgaris* extract: Foliar spray or root drench?. J. Appl. Phycol..

[28] Pennisi G (2019). Resource use efficiency of indoor lettuce (*Lactuca sativa* L.) cultivation as affected by red:blue ratio provided by LED lighting. Sci. Rep..

[29] Baglieri A, Nègre M, Trotta F, Bracco P, Gennari M (2013). Organo-clays and nanosponges for acquifer bioremediation: Adsorption and degradation of triclopyr. J. Environ. Sci. Health B.

[30] La Bella E, Baglieri A, Rovetto EI, Stevanato P, Puglisi I (2021). Foliar spray application of *Chlorella vulgaris* extract: Effect on the growth of lettuce seedlings. Agronomy.

[31] Bradford MM (1976). A rapid and sensitive method for quantitation of microgram quantities of protein utilizing the principle of protein-dye binding. Anal. Biochem..

[32] Miranda KM, Espey MG, Wink DA (2001). A rapid, simple spectrophotometric method for simultaneous detection of nitrate and nitrite. Nitric Oxide.

[33] Kaiser WM, Huber SC (2001). Post-translational regulation of nitrate reductase: Mechanism, physiological relevance and environmental triggers. J. Exp. Bot..

[34] Canovas FM, Canton FR, Gallardo F, Garcia-Gutierrez A, De Vincente A (1991). Accumulation of glutamine synthetase during early development of maritime pine (*Pinus pinaster*) seedlings. Planta.

[35] Avila C, Rotella JR, Canovas FM, De Castro IN, Valpuesta V (1987). Different characteristics of the two glutamate synthetases in green leaves of *Lycopersicon esculentum*. Plant Physiol..

[36] Mulvaney RL, Sparks DL, Page AL, Helmke PA, Loeppert RH, Soltanpour PN, Tabatabai MA, Johnston CT, Sumner ME (1996). Nitrogen—Inorganic forms. Methods of Soil Analysis, Part 3—Chemical Methods.

[37] Baglieri A, Reyneri A, Gennari M, Nègre M (2013). Organically modified clays as binders of fumonisins in feedstocks. J. Environ. Sci. Health B.

[38] Xu G, Fan X, Miller AJ (2012). Plant nitrogen assimilation and use efficiency. Annu. Rev. Plant Biol..

[39] Havlin JL, Beaton JD, Tisdale SL, Nelson WL (1999). Soil Fertility and Fertilizers: An Introduction to Nutrient Management.

[40] Wienhold BJ, Krupinsky JM (1999). Chlorophyll meter as nitrogen management tool in malting barley. Commun. Soil Sci. Plant Anal..

[41] Schepers JS, Francis DD, Vigil M, Below FE (1992). Comparison of corn leaf nitrogen concentration and chlorophyll meter readings. Commun Soil. Sci. Plant Anal..

[42] Peng S, Garcìa FV, Laza RC, Cassman KG (1993). Adjustment for specific leaf weight improves chlorophyll meter’s estimate of rice leaf nitrogen concentration. Agron. J..

[43] Follett RH, Follett RF, Halvorson AD (1992). Use of a chlorophyll meter to evaluate the nitrogen status of dryland winter wheat. Commun. Soil Sci. Plant Anal..

[44] Taiz L, Zeiger E, Møller IM, Murphy A (2018). Plant Physiology and Development.

[45] Brewster JL (1994). Onion and Other Vegetable Alliums.

[46] Abu-Rayyan A, Kharawish BH, Al-Ismail K (2004). Nitrate content in lettuce (*Lactuca sativa* L) heads in relation to plant spacing, nitrogen form and irrigation level. J. Sci. Food Agric..

[47] Thresher A, Foster R, Ponting DJ, Stalford SA, Tennant RE, Thomas R (2020). Are all nitrosamines concerning? A review of mutagenicity and carcinogenicity data. Regul. Toxicol. Pharmacol..

[48] Mirvish SS (1977). N-nitroso compounds, nitrite and nitrate: Possible implications for the causation of human cancer. Prog. Water Technol..

[49] Conversa G, Bonasia A, Lazzizera C, La Rotonda P, Elia A (2021). Reduction of nitrate content in baby-leaf lettuce and *Cichorium endivia* through the soilless cultivation system, electrical conductivity and management of nutrient solution. Front. Plant Sci..

[50] EFSA Opinion of the Scientific Panel on Contaminants in the Food chain on a request from the European Commission to perform a scientific risk assessment on nitrate in vegetables. *EFSA Journal* **689**, 1–79; http://www.efsa.europa.eu/en/scdocs/doc/689.pdf (2008).

[51] EFSA Panel on Contaminants in the Food Chain (CONTAM) Scientific Opinion on possible health risks for infants and young children from the presence of nitrates in leafy vegetables. *EFSA Journal* **8**(12), www.efsa.europa.eu/efsajournal.htm (2010) 10.2903/j.efsa.2010.1935 PMC13085130 42004122

[52] Cedergreen N, Madsen TV (2003). Nitrate reductase activity in roots and shoots of aquatic macrophytes. Aquat. Bot..

[53] Nemie-Feyissa D, Królicka A, Førland N, Hansen M, Heidari B, Lillo C (2013). Post-translational control of nitrate reductase activity responding to light and photosynthesis evolved already in the early vascular plants. J. Plant Physiol..

[54] Pinto E, Fidalgo F, Teixeira J (2014). Influence of the temporal and spatial variation of nitrate reductase, glutamine synthetase and soil composition in the N species content in lettuce (*Lactuca sativa*). Plant Sci..

[55] Lea PJ, Leegood RC (1993). Nitrogen metabolism. Plant Biochemistry and Molecular Biology.

[56] Gupta N, Gupta AK, Gaur VS, Kumar A (2012). Relationship of nitrogen use efficiency with the activities of enzymes involved in nitrogen uptake and assimilation of finger millet genotypes grown under different nitrogen inputs. Sci. World J..

[57] Puglisi I, Barone V, Fragalà F, Stevanato P, Baglieri A, Vitale A (2020). Effect of microalgal extracts from *Chlorella vulgaris* and *Scenedesmus quadricauda* on germination of *Beta vulgaris* seeds. Plants.

[58] Ottaiano L, Di Mola I, Cozzolino E, El-Nakhel C, Rouphael Y, Mori M (2021). Biostimulant application under different nitrogen fertilization levels: Assessment of yield, leaf quality, and nitrogen metabolism of tunnel-grown lettuce. Agronomy.

[59] Navarro-León E, López-Moreno FJ, Borda E, Marín C, Sierras N, Blasco B, Ruiz JM (2022). Effect of l -amino acid-based biostimulants on Nitrogen Use Efficiency (NUE) in lettuce plants. J. Sci. Food Agric..

[60] Chilom G, Baglieri A, Johnson-Edler CA, Rice JA (2013). Hierarchical self-assembling properties of natural organic matter’s components. Org. Geochem..

[61] Puglia D, Pezzolla D, Gigliotti G, Torre L, Bartucca ML, Del Buono D (2021). The Opportunity of valorizing agricultural waste, through its conversion into biostimulants, biofertilizers, and biopolymers. Sustainability.

[62] Mghaiouini R (2022). Formulation of new biostimulant of plant and soil correction based on humic acids extracted by magnetized water from compost from the waste of coffee marc and cattle manure. Waste Biomass Valor..

[63] Lemaire G, Ciampitti I (2020). Crop mass and N status as prerequisite covariables for unraveling nitrogen use efficiency across genotype-by-environment-by-management scenarios: A review. Plants.

